# Glucose Sensing in L Cells: A Primary Cell Study

**DOI:** 10.1016/j.cmet.2008.11.002

**Published:** 2008-12-06

**Authors:** Frank Reimann, Abdella M. Habib, Gwen Tolhurst, Helen E. Parker, Gareth J. Rogers, Fiona M. Gribble

**Affiliations:** 1 Cambridge Institute for Medical Research and Department of Clinical Biochemistry, Addenbrooke's Hospital, Hills Road, Cambridge CB2 0XY, UK

**Keywords:** HUMDISEASE

## Abstract

Glucagon-like peptide-1 (GLP-1) is an enteric hormone that stimulates insulin secretion and improves glycaemia in type 2 diabetes. Although GLP-1-based treatments are clinically available, alternative strategies to increase endogenous GLP-1 release from L cells are hampered by our limited physiological understanding of this cell type. By generating transgenic mice with L cell-specific expression of a fluorescent protein, we studied the characteristics of primary L cells by electrophysiology, fluorescence calcium imaging, and expression analysis and show that single L cells are electrically excitable and glucose responsive. Sensitivity to tolbutamide and low-millimolar concentrations of glucose and α-methylglucopyranoside, assessed in single L cells and by hormone secretion from primary cultures, suggested that GLP-1 release is regulated by the activity of sodium glucose cotransporter 1 and ATP-sensitive K^+^ channels, consistent with their high expression levels in purified L cells by quantitative RT-PCR. These and other pathways identified using this approach will provide exciting opportunities for future physiological and therapeutic exploration.

## Introduction

Enteroendocrine L cells secrete a number of physiologically important peptides, including glucagon-like peptide-1 (GLP-1), glucagon-like peptide-2 (GLP-2), peptide YY (PYY) and oxyntomodulin. Each of these hormones is under evaluation for potential therapeutic applications in humans. GLP-1 mimetics and inhibitors of GLP-1 degradation are already licensed for the treatment of type 2 diabetes, exploiting the important role of GLP-1 in the stimulation and maintenance of insulin release from pancreatic β cells and in the control of appetite (Drucker and Nauck, 2006; Holst, 2007). GLP-2, by contrast, enhances regeneration and repair of the intestinal epithelium (Estall and Drucker, 2006), whereas PYY and oxyntomodulin reduce food intake in rodents and humans (Wynne and Bloom, 2006). Targeting secretion from the L cell, therefore, provides exciting new therapeutic opportunities, and a more detailed characterization of the molecular and cellular physiology of this cell type is urgently needed to fuel the future progress of this research field.

L cells form a component of the diffuse enteroendocrine system, which includes a variety of endocrine cell types scattered along the length of the intestinal epithelium. To date, the dispersed nature of enteroendocrine cells has largely precluded their interrogation by single-cell physiological techniques. Histamine-producing enterochromaffin-like cells, which can be partially purified and maintained in short-term culture, are regulated by voltage-dependent calcium entry but are not believed to be electrically active, as they lack voltage-gated sodium currents (Bufler et al., 1998). For most other enteroendocrine cell types, single-cell characterization has been restricted to the study of model cell lines. GLUTag, STC-1, and NCI-H716 cells secrete GLP-1 (Drucker et al., 1994; Abello et al., 1994; Reimer et al., 2001) but exhibit different nutrient sensitivities and are incompletely validated as accurate models of the native L cell. While GLP-1 release has also been studied in primary cultures of fetal rat intestinal cells (Brubaker and Vranic, 1987), establishing cultures from adult intestinal tissue has presented more of a challenge.

GLP-1 release is triggered by ingestion of carbohydrates, fats, and protein and is believed to reflect, at least in part, the direct sensing of luminal nutrients by the apical processes of the L cells. In GLUTag cells, we previously identified two distinct pathways that couple GLP-1 secretion to glucose exposure, one involving the classical glucose-sensing machinery employed by the pancreatic β cell, mediated through glucose metabolism and closure of ATP-sensitive potassium (K_ATP_) channels, and the other exploiting the electrogenic nature of Na^+^-coupled glucose uptake by sodium glucose cotransporters (SGLTs) (Reimann and Gribble, 2002; Gribble et al., 2003). However, subunits of the taste receptor pathway (Tas1 receptors and α-gustducin) have also been detected in intestinal cells, including some cells that coexpress GLP-1 (Sutherland et al., 2007), and recent evidence suggests that they may contribute to the stimulation of GLP-1 release and the upregulation of intestinal SGLT1 expression following sugar ingestion (Jang et al., 2007; Margolskee et al., 2007).

To enable a molecular and single-cell characterization of GLP-1 secretory mechanisms, we made a transgenic mouse model in which cells expressing proglucagon were labeled by the yellow fluorescent protein, Venus (Nagai et al., 2002). This was used to interrogate components of the stimulus-secretion coupling pathways underlying GLP-1 release, using electrophysiological recordings and fluorescence-based calcium measurements from identified adult murine L cells in primary culture and supported by expression analysis and measurements of hormone secretion from primary intestinal cultures.

## Results

Pronuclear injection of BAC constructs containing the *Venus* sequence in the place of the coding region of *proglucagon* (described in Supplemental Experimental Procedures and Figure S1 available online) resulted in the generation of five founder mice with 1 to 12 copies of the transgene, as assessed by quantitative PCR. Sporadic yellow fluorescent cells were observed in freshly harvested tissue from the gastrointestinal tract, pancreatic islets, and brainstem of the transgenic mice, coinciding with the known locations of GLP-1 and glucagon expression (Larsson et al., 1975; Larsen et al., 1997). As we observed no differences between the strains in the global pattern of Venus expression in the gut and pancreas, the five strains were used interchangeably for the remainder of the study.

**Figure 1:**
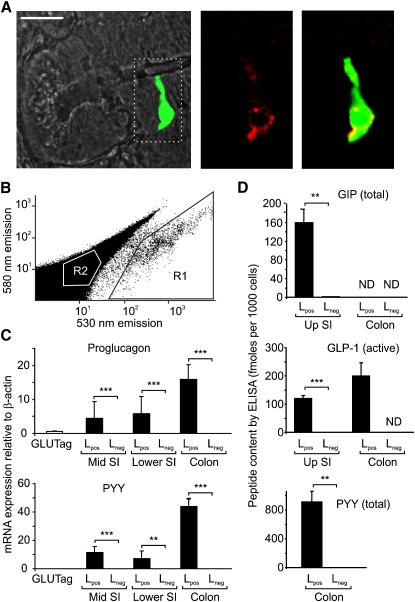
Cell-Specific Venus Expression in Transgenic Mice (A) Colocalization of Venus fluorescence (green) with glucagon immunofluorescence (red) in the small intestine. Scale bar, 20 μm. (B) L cells were collected by FACS sorting with gates on pulse width, side and forward scatter to select single cells and yellow (580 nm) and green (530 nm) fluorescence to select Venus-positive (R1) or Venus-negative (R2) cells (excitation 488 nm). The figure shows a representative sort from the small intestine. (C) Relative expression of *proglucagon* and *Pyy* mRNAs in GLUTag cells and L_pos_ and L_neg_ cells from different regions of the intestine (middle third of small intestine [mid S1], lower third of small intestine [lower S1], and colon). Data are presented as geometric mean and upper SE (n ≥ 3 each). ^∗∗^p < 0.01, ^∗∗∗^p < 0.001 by Student's t test. (D) GIP, GLP-1, and PYY peptide contents in L_pos_ and L_neg_ cells from the small intestine (upper half, Up SI) or colon, as indicated, collected by FACS sorting and measured by ELISA. Data represent mean and SE of ≥ 3 samples with statistical comparisons between corresponding L_pos_ and L_neg_ cells. ^∗∗^p < 0.01, ^∗∗∗^p < 0.001. ND, not detected.

In the gut, Venus-positive cells increased in density along the intestinal axis from the duodenum to the colon in agreement with previous descriptions of GLP-1-positive cells identified by immunohistochemistry (Larsson et al., 1975). The cells were located in both crypts and villi and were frequently observed to have thin processes extending to the gut lumen. In all strains, Venus fluorescence in the intestine coincided with immunofluorescence staining for proglucagon (Figure 1A). *Proglucagon* gene expression was 11,000-fold higher, and GLP-1 protein content was >7000-fold higher in Venus-positive cells (L_pos_) purified by FACS (Figure 1B) than in nonfluorescent cells collected in parallel (L_neg_), thus confirming the cell specificity of expression of the fluorescent transgene (Figures 1C and 1D). Consistent with previous reports, L_pos_ cells in the gut also produced PYY, detectable at the mRNA and protein levels as well as by immunofluorescence staining (Figures 1C, 1D, and S1B). GIP mRNA (data not shown) and protein (Figure 1D) was found in L cells from the small intestine only, although their GIP protein content was 6-fold lower than that of K cells purified by a similar method (F.R., unpublished data). These results are compatible with previous reports that GIP is detectable by immunostaining in a subset of small intestinal L cells (Mortensen et al., 2003).

In pancreatic islets, Venus fluorescence colocalized with proglucagon immunostaining, confirming the identity of the labeled cells as glucagon-producing α cells (Figure S2A). Islet cells from the transgenic mice could be separated by flow cytometry into relatively pure populations of α and β cells and a mixed population of δ and pancreatic polypeptide (PP) cells, as assessed by their hormone expression profiles measured by quantitative RT-PCR (Figures S2B and S2C).

### Primary Intestinal Cultures

As FACS-sorted single L cells did not survive in culture, we generated mixed primary cultures from adult mouse small intestine or colon, which could be maintained for up to 2 weeks. Venus-positive cells in the cultures were immunopositive for glucagon and PYY (Figure S1D). Cell proliferation, assessed by EdU incorporation, was evident in the nonfluorescent cell population, but not in the 154 L_pos_ cells examined (Figure S1E). In 8-day-old colonic cultures, forskolin plus isobutyl-1-methylxanthine (IBMX, 10 μM of each) enhanced GLP-1 release 9-fold and PYY release 6-fold (n = 3 of each, data not shown), indicating that the L cells in culture were functionally viable.

### Electrical Activity in Colonic L Cells

**Figure 2:**
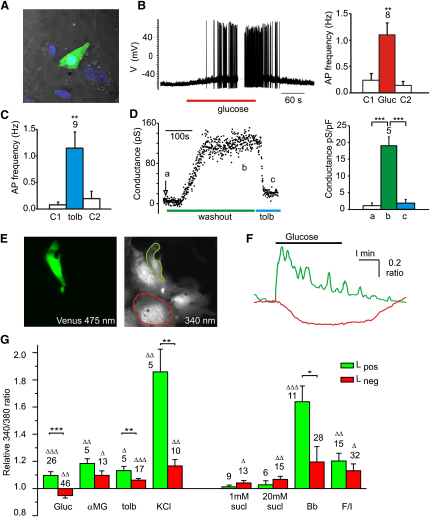
Electrical Activity and [Ca^2+^]_i_ in Cultured Colonic L Cells (A) Colonic epithelial cells fixed after 10 days in primary culture. DIC, DAPI (blue), Venus (green). (B) Glucose-triggered electrical activity in L cells. (Left) Representative whole-cell perforated patch current clamp recording of a Venus-positive cell stimulated with glucose (10 mM) applied as indicated. (Right) Average action potential frequency before (C1), during (Gluc), and after (C2) application of glucose measured in eight cells. Error bars represent 1 SE and significance, between AP-frequency in the presence and absence of glucose was tested by Student's paired t test. ^∗∗^p < 0.01. (C) Average action potential frequency before (C1), during (tolb), and after (C2) application of tolbutamide (500 μM) measured in nine cells, as in (B). Error bars represent 1 SE, and significance between AP-frequency in the presence and absence of tolbutamide was tested by Student's paired t test. ^∗∗^p < 0.01. (D) Functional K_ATP_ wash-out currents in cultured L cells. (Left) Slope conductances between −70 and −50 mV were recorded from a cell in conventional whole-cell voltage clamp. The arrow indicates the time when the cell attached mode was converted into conventional whole cell with 300 μM ATP in the pipette. Tolbutamide (tolb, 500 μM) was applied at steady state, as indicated. (Right) Average conductances measured as exemplified on the left in five cells at times (a) cell attached, (b) wash-out steady state, and (c) after application of tolbutamide. Error bars represent 1 SE, and significance was tested by Student's paired t test. ^∗∗∗^p < 0.001. (E) L_pos_ and L_neg_ cells in colonic cultures were loaded with fura2-AM and identified by their presence/absence of Venus fluorescence (475 nm excitation, left). The image of fura2-loaded cells excited at 340 nm (right) was used to outline L_pos_ (green) and L_neg_ (red) cells. (F) The 340/380 nm fluorescence ratios (reflecting [Ca^2+^]_i_) of the two cells outlined in (E) are shown following addition of 10 mM glucose to the perfusate. Green trace, L_pos_ cell; red trace, L_neg_ cell. (G) Mean calcium changes in L_pos_ (green bars) and L_neg_ (red bars) cells, identified and monitored as in (E) and (F), following the addition of glucose (Gluc, 10 mM), αMG (10 mM), tolbutamide (tolb, 100 μM), KCl (30 mM), sucralose (sucl, 1 or 20 mM), bombesin (Bb, 100 nM), and forskolin/IBMX (F/I, 10 μM of each), as indicated. 340/380 ratios in the presence of the test agent were normalized to the mean of the background ratios of each cell measured before addition and after washout of the test compound. Data represent the mean and SE of the number of cells indicated above each bar. Δp < 0.05, ΔΔp < 0.01, and ΔΔΔp < 0.001 compared with baseline. ^∗^p < 0.05, ^∗∗^p < 0.01, and ^∗∗∗^p < 0.001 for comparison between corresponding L_pos_ and L_neg_ cells by Student's t test.

L cells were readily distinguishable in culture by their Venus fluorescence (Figure 2A). In perforated patch recordings, they were electrically excitable and exhibited an increase in action potential frequency following addition of glucose or the sulphonylurea tolbutamide (Figures 2B and 2C). Conventional whole-cell recordings with a low pipette ATP concentration (0.3 mM) revealed a tolbutamide-sensitive current that developed over the course of 2–5 min, confirming the presence of functional K_ATP_ channels in primary L cells (Figure 2D).

### Calcium Responses in Colonic L Cells

Cultured L cells, identified by their Venus fluorescence, exhibited intracellular calcium ([Ca^2+^]_i_) elevations (Figures 2E, 2F, and 2G) in response to glucose (10 mM), α-methylglucopyranoside (αMG, 10 mM), tolbutamide (100 μM), KCl (30 mM), forskolin/IBMX (10 μM of each), or bombesin (100 nM), but not to the artificial sweetener sucralose (1 mM or 20 mM). L_neg_ cells were significantly less responsive than L cells to glucose, tolbutamide, KCl, and bombesin, and a proportion of L_neg_ cells, but not L_pos_ cells, responded to sucralose. The significantly greater effects of glucose and tolbutamide on L_pos_ cells than on L_neg_ cells suggest that glucose responsiveness originates in the L cells themselves.

### GLP-1 Secretion from Primary Cultures

**Figure 3:**
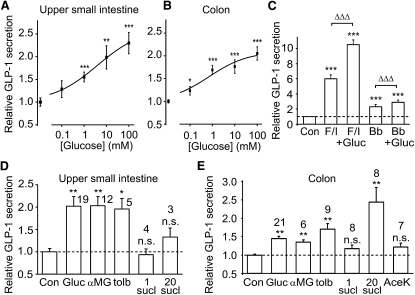
GLP-1 Secretion from Primary Intestinal Cultures (A and B) Mixed primary cultures from the upper half of the small intestine (A) or the colon (B) were incubated in bath solution containing the glucose concentrations indicated. The percentage of GLP-1 secretion in each well is expressed relative to the basal secretion measured in parallel on the same day. Dose-response curves were fitted through the entire data set using a logistic equation, giving EC_50_s of 4 mM and 0.7 mM for the small intestine and colon, respectively (not significantly different). Error bars represent 1 SE, and significance is shown relative to baseline using a single-factor t test. ^∗^p < 0.05, ^∗∗^p < 0.01, ^∗∗∗^p < 0.001. (C) GLP-1 secretion from primary colonic cultures triggered by forskolin/IBMX (F/I, 10 μM of each), bombesin (Bb, 100 nM), or glucose (Gluc, 10 mM), as indicated, measured and expressed as in (A). Error bars represent 1 SE, and significance is shown relative to baseline using a single-factor t test: ^∗∗∗^p < 0.001. The additional effect of glucose was analyzed by regression analysis to compensate for variation in the effectiveness of forskolin/IBMX or bombesin between different experiments: ΔΔΔp < 0.001. (D and E) GLP-1 secretion from the upper small intestine (D) or colon (E), measured as in (A), following addition of glucose (Gluc, 10 mM), αMG (10 mM), tolbutamide (tolb, 500 μM), sucralose (sucl, 1 or 20 mM), or acesulfame K (AceK, 2 mM). The control bar (Con) and dashed line indicate the basal rate of secretion. Data represent the mean and SE of the number of wells indicated, and significance is shown relative to baseline, tested by a single-factor t test. ^∗^p < 0.05, ^∗∗^ p < 0.01. n.s., not significant.

Although glucose-triggered GLP-1 release has been well documented in vivo, as well as in whole intestinal preparations and GLUTag cells (Herrmann et al., 1995; Plaisancié et al., 1995; Reimann and Gribble, 2002), a lack of glucose responsiveness of fetal rat intestinal cultures and primary cultured canine L cells (Brubaker and Vranic, 1987; Damholt et al., 1998) has led to doubts about whether L cells are themselves glucose sensitive. In primary cultures of small intestine and colon, however, GLP-1 secretion was glucose dependent, with EC_50_s of 4 mM and 0.7 mM, respectively (not significantly different, Figures 3A and 3B). To examine the relative roles of SGLT1 and K_ATP_ channels in L cells, we measured GLP-1 responses to the SGLT1 substrate, αMG, or to tolbutamide (Figures 3C and 3D). GLP-1 release was triggered by αMG with a measured EC_50_ in the small intestine of 0.2 mM (data not shown), not significantly different than that for glucose and similar to the K_m_ for SGLT1 of 0.3 mM (Díez-Sampedro et al., 2000). Secretion was also stimulated by tolbutamide, confirming a functional role for K_ATP_ channels. By contrast, GLP-1 secretion was not triggered by 1 mM sucralose or 2 mM acesulfame K, agents that specifically target sweet taste receptors (Figures 3C and 3D). At a higher concentration of 20 mM, sucralose increased GLP-1 release from colonic cultures, but not small intestinal cultures, an effect that was additive with glucose and therefore likely to act via an independent pathway (Figure S3). Overnight pretreatment of colonic cultures with 20 mM sucralose did not affect either basal GLP-1 release or the subsequent response to 10 mM glucose (Figure S3).

### Expression of Candidate Glucose-Sensing Machinery in Purified L and Islet Cells

**Figure 4:**
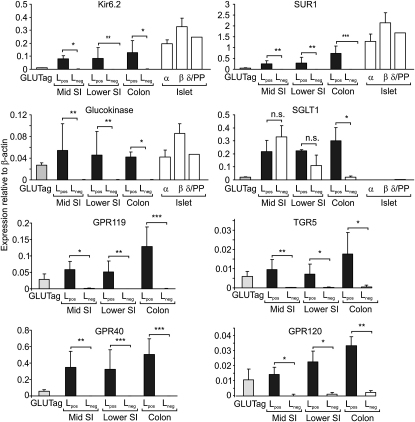
Expression Analysis Expression of the genes indicated in GLUTag cells, L_pos_ cells, and L_neg_ cells from three different regions of the gut (middle third of small intestine [SI], lower third of small intestine, and colon) and in pancreatic α, β, and δ/PP cells isolated as in Figure S2. Expression was normalized to that of *β-actin* in the same sample. Data are presented as geometric mean and upper SE (n ≥ 3 each). L_pos_ and L_neg_ cells were compared by Student's t tests: ^∗^p < 0.05, ^∗∗^p < 0.01, ^∗∗∗^p < 0.001. n.s., not significant.

To confirm the identity and expression of candidate glucose-sensing components in L cells, FACS-sorted L_pos_ and L_neg_ cells were analyzed by quantitative RT-PCR, using probe sets against K_ATP_ channel subunits (*Kir6.2*, *Sur1*), glucokinase (*Gck*), facilitative glucose transporters (*Glut1*, *2*, *3*, and *5*), sodium-coupled glucose transporter 1 (*Sglt1*), taste receptor subunits (*Tas1R1*, *R2*, and *R3*), and *α-gustducin*. Comparisons were made with expression levels in GLUTag and STC-1 cells, different islet cell populations, and tongue epithelium (Figure 4 and Table S1).

#### K_ATP_ Channels and Glucokinase

L cells expressed *Kir6.2*, *SUR1*, and glucokinase (*Gck*) at levels that were enhanced several hundred-fold compared with Venus-negative controls and with no apparent differences along the intestinal axis. Expression levels were comparable with those found in pancreatic α, β, and δ/PP cells. *Kir6.2* expression was lower even in STC-1 cells than in GLUTag cells (p = 0.002), consistent with a previous report that hormone secretion from an STC-1 subclone was K_ATP_ channel independent (Wang et al., 2003).

#### Facilitative Glucose Transporters

*Glut1* expression was similar in L_pos_ and L_neg_ cells and increased from the midsmall intestine to colon, whereas *Glut2* expression was highest proximally and decreased along the gut axis in both L_pos_ and L_neg_ cells. *Glut5* was abundant in small intestinal L_neg_ cells and all L_pos_ cell populations examined but was 70-fold lower in colonic L_neg_ than colonic L_pos_ cells. In pancreatic islets, *Glut2* expression was 1200-fold higher and *Glut5* 5-fold higher in β than α cells, whereas both *Glut1* and *Glut3* were expressed at similar low levels in both cell types.

#### Sodium-Coupled Glucose Transporters

*Sglt1* was highly expressed in both L_pos_ and L_neg_ cells from the small intestine, consistent with its known role in epithelial glucose absorption, whereas, in the colon, we observed a 9-fold lower level of expression in L_neg_ than L_pos_ cells (p = 0.002). Immunofluorescence staining localized SGLT1 to the apical membrane of ileal enterocytes and L cells (Figure S1C), and, in colonic cultures, the predominance of SGLT1 in L_pos_ compared with L_neg_ cells was also evident by immunostaining (Figure S1F). *Sglt1* expression was barely detectable in the islets.

#### Sweet Taste Receptors

The taste receptor subunit *Tas1R3* was expressed at low levels in both the intestine and islets with no apparent difference between L_pos_ and L_neg_ cells (Table S1). *Tas1R2* expression was generally lower but could be detected in colonic and lower small intestinal L_pos_ cells as well as pancreatic α cells. *α-Gustducin* expression was detectable in pancreatic α cells and colonic L_pos_ cells but was below the detection limit in most other cell preparations tested. Although low, the expressions of the *Tas1* receptors and *α-gustducin* are comparable with those measured in the tongue epithelium, which is known to include a limited proportion of taste-responsive cells.

### Expression and Function of G Protein-Coupled Receptor Pathways in L Cells

The ability of elevated cAMP or bombesin (G_q_ coupled) to increase [Ca^2+^]_i_ in L cells was shown above. These pathways were also examined by measuring GLP-1 secretion from primary cultures (Figure 3E). GLP-1 release was triggered by forskolin/IBMX or bombesin and was further enhanced by glucose, indicating a potential synergistic interaction between depolarizing stimuli and signaling pathways activated by G protein-coupled receptors.

Lipids have been reported recently to stimulate L cells via G protein-coupled receptors. The G_αs_-coupled receptor GPR119 is an orphan G protein coupled receptor responsive to the natural ligand oleoylethanolamide (Overton et al., 2006) and is under evaluation as a therapeutic target in L cells (Chu et al., 2008). The long chain fatty acid receptors GPR120 and GPR40 and the bile acid receptor TGR5 have also been shown to play functional roles in enteroendocrine cell lines (Hirasawa et al., 2005; Katsuma et al., 2005). mRNAs for all of these GPCRs were strongly expressed in FACS-sorted L cells (Figure 4).

## Discussion

By fluorescently labeling cells that express proglucagon in a transgenic mouse, we show that primary L cells are electrically excitable and directly nutrient responsive. The fluorescently tagged L cells are suitable for patch clamping, single-cell dynamic calcium imaging, and cell sorting, providing a range of new and powerful techniques to interrogate the function of this enteroendocrine cell type. Our results support the idea that certain nutrients like glucose can directly stimulate L cells by triggering a cascade of membrane depolarization, action potential firing, and voltage-dependent Ca^2+^ entry. Furthermore, as the electrical activity and glucose responsiveness of primary mouse L cells mimicked our previous findings on the GLUTag cell line (Reimann and Gribble, 2002), our data support the validity of using this cell line as a model for the native L cell.

The low millimolar EC_50_ for glucose-triggered GLP-1 release from primary intestinal cultures (0.7–4 mM) is similar to that of GLUTag cells (0.2 mM) (Gribble et al., 2003) and close to the reported K_m_ for SGLT1 of 0.3 mM (Díez-Sampedro et al., 2000). Indeed, the finding that the nonmetabolizable glucose analog αMG also triggered GLP-1 release with an EC_50_ of 0.2 mM suggests that SLGT1 is itself an L cell glucose sensor. By contrast, GLUTag cells were only responsive to αMG at 100 mM, consistent with the 11-fold lower expression of *Sglt1* mRNA in the cell line compared with small intestinal L_pos_ cells. SGLT1 activity underlies a major component of apical membrane glucose uptake in small intestinal enterocytes, utilizing the inwardly directed sodium gradient to drive glucose influx even at low luminal glucose concentrations. Our results support previous reports that the ability of luminal sugars to stimulate GLP-1 release was both sodium dependent and matched the sugar specificity of the intestinal glucose uptake pathway (Herrmann et al., 1995; Ritzel et al., 1997). By contrast, the function of facilitative glucose transporters in L cells remains unclear, although mice lacking GLUT2 had a lower intestinal GLP-1 content and reduced plasma GLP-1 concentrations after an oral glucose tolerance test (Cani et al., 2007). If localized on the basolateral surface as in enterocytes, they could provide an important glucose efflux pathway as well as a potential route to respond to plasma glucose changes. Although GLP-1 release is not stimulated by intravenous glucose administration in fasting human subjects, it was strongly influenced by the plasma glucose concentration in a perfused porcine ileum preparation with a constant flow of luminal glucose (Hansen et al., 2004).

L cells express *glucokinase* and have functional K_ATP_ channels, supporting a recent observation that Kir6.2 was detected in human L cells by immunostaining (Nielsen et al., 2007). The role of K_ATP_ channels in L cells is unclear, but it is possible that they might be modulated by basolateral glucose and/or neuronal inputs. As SGLT1-associated currents are relatively small, their effectiveness would be determined by the magnitude of other plasma membrane currents. Alterations of K_ATP_ channel activity by metabolism or neurotransmitters might, therefore, provide a route to regulate the sensitivity of an L cell to luminal glucose.

Although recent reports suggest that sweet taste receptor activation stimulates GLP-1 secretion and increases intestinal SGLT1 expression (Jang et al., 2007; Margolskee et al., 2007), this is unlikely to explain our finding that GLP-1 release from primary cultures was triggered by submillimolar glucose concentrations, as sweet taste receptors are typically activated by 30–1000 mM glucose and by low millimolar concentrations of sucralose and acesulfame K (Damak et al., 2003). We were unable to demonstrate an effect of 1 mM sucralose on either GLP-1 release or [Ca^2+^]_i_ in L cells, consistent with a recent report that sweeteners do not stimulate GLP-1 release in vivo in rats (F. Fujita et al., 2008, American Diabetes Association, abstract). GLP-1 release from colonic cultures was, however, triggered by a relatively high concentration of sucralose (20 mM), an effect that was additive with glucose. Although we are unable to conclude whether the action of the higher concentration of the sweetener is mediated by the sweet taste receptor pathway, colonic L_pos_ cells did have detectable levels of *Tas1R2*, *Tas1R3*, and *α-gustducin* expression, in agreement with previous reports that a small subpopulation of GLP-1-positive cells also express α-gustducin (Sutherland et al., 2007; Jang et al., 2007). In taste buds, the coupling of sweet taste receptors to α-gustducin and phospholipase Cβ2 results in elevated [Ca^2+^]_i_ and/or cAMP (Margolskee, 2002). The lack of responsiveness of [Ca^2+^]_i_ in cultured L cells to sucralose does not appear to reflect a defect in potential downstream signaling pathways, as rapid [Ca^2+^]_i_ changes in L cells and GLP-1 secretion were triggered by forskolin/IBMX or bombesin.

Glucose-triggered GLP-1 release was amplified by forskolin/IBMX and additive with the response to bombesin. The potent effects of elevated cAMP and G_q_-coupled receptor activation, together with the L cell-specific expression of receptors such as *Gpr40*, *Gpr120*, *Gpr119*, and *Tgr5* in the intestine, make these pathways exciting targets for the future development of therapeutic L cell secretagogues. The availability of transgenic mice with fluorescently labeled L cells paves the way for a new wave of exploration into the mechanisms underlying GLP-1 and PYY release, with the potential to identify targets in L cells that could be exploited therapeutically for the treatment of diabetes and obesity.

## Experimental Procedures

### Constructs

BAC constructs containing the sequence of *Venus* driven by the rat and mouse proglucagon promoters were made by the RedEt technique (Zhang et al., 2000). For further details, see the Supplemental Data.

### Intestinal Epithelial Cell Isolation

All animal procedures were approved by the local ethical committee and conformed to Home Office regulations. The 2- to 6-month-old Venus-expressing transgenic mice were sacrificed by cervical dislocation and the gut collected into ice-cold Leibovitz-15 (L-15) medium (PAA, UK). The small intestine (from the stomach to ileocaecal junction) was divided either into thirds (top, mid, and lower) or into halves, as indicated in the text, and colonic samples consisted of gut tissue distal to the ileocolic junction. The intestine was opened, rinsed in L-15, and chopped into 1–2 mm pieces. For flow cytometry, tissue was digested twice for 30 min with 1 mg/ml Collagenase-XI in calcium-free Hanks Balanced Salt Solution (HBSS) at 37°C. Resulting cell suspensions were filtered through 70 μm nylon cell strainers (BD Falcon, UK), centrifuged at 300 × g for 5 min, and pellets resuspended in L-15 supplemented with 10% fetal bovine serum (FBS). For culture, tissue was digested with 0.4 mg/ml Collagenase XI, centrifuged at 300 × g, and resuspended in Dulbecco's modified Eagle's medium (25 mM glucose) supplemented with 10% FBS, 2 mM L-glutamine, penicillin, and streptomycin. Aliquots were plated on matrigel-coated 24-well plates, 35 mm plastic dishes, or glass-bottom culture dishes (Mattek Corporation, USA) for secretion, electrophysiology, and imaging studies, respectively, and incubated for 1–12 days at 37°C, 5% CO_2_.

### Flow Cytometry

A MoFlo Beckman Coulter Cytomation sorter (488 nm excitation) was used to separate populations of > 95% pure Venus-positive or -negative cells. Settings: 90 μm nozzle, 38–40 psi, 58–60 kHz, plates charged with 3200 V. Single cells were selected by their side and forward scatter and pulse width. Venus-positive cells were selected by their relative fluorescence at 530 and 580 nm, and Venus-negative cells by a gate that excluded Venus fluorescence. Cells were sorted at numbers of up to 30,000 into either RNAlater (Ambion, UK) or lysis buffer for protein extraction (see below).

### RNA Extraction and Quantitative RT-PCR

Total RNA from FACS-sorted cells was isolated using a micro scale RNA isolation kit (Ambion) and reverse transcribed according to standard protocols. Quantitative RT-PCR was performed with 7900 HT Fast Real-Time PCR system (Applied Biosystems). The PCR reaction mix consisted of first-strand cDNA template, primer pairs (for details, see Supplemental Data), 6-carboxyfluorescein/quencher probes (Bioresearch Technologies, CA and Applied Biosystems), and PCR Master mix (Applied Biosystems). In all cases, expression was compared with that of *β-actin* measured on the same sample in parallel on the same plate, giving a CT difference (ΔCT) for *β-actin* minus the test gene. CTs for *β-actin* ranged from 20–25 in the different samples, and reactions in which the test gene was undetectable were assigned a CT value of 40. A ΔCT value of −12 to −15 is, therefore, close to the detection limit of the assay. Mean, standard error, and statistics were performed on the ΔCT data and only converted to relative expression levels (2ˆΔCT) for presentation in the figures.

### Hormone Secretion

Secretion studies were performed 24–36 hr after plating using tissue from either transgenic or nontransgenic mice, as preliminary experiments showed that the transgene had no effect on GLP-1 secretion (data not shown). Cultures were incubated with test reagents in bath solution (see below) containing 0.1% fatty acid-free BSA for 2–4 hr at 37°C. Media was collected and centrifuged to remove contaminating cells. Cells were then treated with lysis buffer containing: 50 mM Tris-HCl, 150 mM NaCl, 1% IGEPAL-CA 630, 0.5% deoxycholic acid, and one tablet of complete EDTA-free protease inhibitor cocktail (Roche) to extract intracellular peptides. GLP-1 was assayed in supernatants and cell extracts using an active GLP-1 ELISA-kit (Millipore, USA); PYY was assayed using a total peptide YY ELISA kit (Diagnostic Systems Laboratories, Inc., USA) and GIP by a rodent GIP total ELISA kit (Millipore, USA). Hormone secretion was expressed as a fraction of the total of that hormone measured in each well and normalized, as indicated, to the basal secretion measured in parallel on the same day.

### Calcium Imaging

Experiments were performed on 3- to 12-day-old cultures. Cells were loaded in 7 μM fura2-AM (Invitrogen, UK) and 0.01% pluronic F127 and incubated in standard bath solution containing 10 mM glucose and 300 μM eserine for 30 min at 37°C. Experiments were performed using an inverted fluorescence microscope (Eclipse TE2000, Nikon, UK) with a 40× oil-immersion objective. Fura2 was excited at 340 and 380 nm and Venus at 475 nm using a 75 W xenon arc lamp and a monochromator (Cairn Research, Faversham, UK) controlled by MetaFluor software (Molecular Devices, UK). Emission was recorded with a QuantEM CCD camera (Photometrics, Roper Scientific, UK). Fura2 fluorescence measurements were taken every 2 s, background corrected, and expressed as the 340/380 nm ratio. Solutions were perfused at ∼1 ml/min, and test agents were added for 2–5 min. Average fluorescence ratios were determined over 20 s periods under control conditions (before addition of and after washout of the test agent) and at the time of the maximum response during application of the agent.

### Electrophysiology

Experiments were performed on 3- to 12-day-old colonic cultures at 22–25°C using techniques and pipette solutions described previously for the study of GLUTag cells (Reimann and Gribble, 2002; Simpson et al., 2007). Slope conductance was calculated for the linear part of the current-voltage relationship between −70 to −50 mV.

### Solutions

Standard bath solution contained (mM) 4.5 KCl, 138 NaCl, 4.2 NaHCO_3_, 1.2 NaH_2_PO_4_, 2.6 CaCl_2_, 1.2 MgCl_2_, and 10 HEPES (pH 7.4, NaOH). All chemicals were supplied by Sigma Aldrich (Poole, UK) unless otherwise stated.

### Immunohistochemistry

See Supplemental Data.

### Data Analysis

Comparisons involving multiple conditions were performed initially either by ANOVA or regression analysis and, subsequently, by Student's two-sample or one-sample t tests (Microsoft Excel), as indicated, with a threshold for significance of p = 0.05. Dose-response curves for glucose-triggered GLP-1 release were fit with a logistic equation and compared using a comparison of fits analysis using Microcal Origin software.

## Supplemental Data

Document S1. Supplemental Experimental Procedures, Three Figures, and Three Tables
