# Supplementary material for: Glucose Sensing in L Cells: A Primary Cell Study
Source: Cell Metab. 2008 Dec 6;8(6-3):532–9. doi: 10.1016/j.cmet.2008.11.002 (PMC2697331; doi:10.1016/j.cmet.2008.11.002)
Supplement: Document S1. Supplemental Experimental Procedures, Three Figures, and Three Tables [file mmc1.pdf]

Cell Metabolism, *Volume 8*

Supplemental Data

Glucose Sensing in L Cells: A Primary Cell Study

Frank Reimann, Abdella M. Habib, Gwen Tolhurst, Helen E. Parker, Gareth J. Rogers, and Fiona M. Gribble

Supplementary Methods, Figure Legends, and Tables

Supplementary Methods

Creation of Proglucagon-Promoter Driven Fluorescence Protein Expressing Transgenic Mice

To express a modified yellow fluorescent protein (YFP-Venus, gift of A. Miyawaki) under the control of the proglucagon promoter, two BAC constructs were created using Red/ET recombination technology. One construct was based on rat genomic sequence starting from the BAC CH230-36N4 (Children's Hospital Oakland Research Institute, CHORI), the second construct started from the BAC RP23-343C17 (CHORI), which contains mouse genomic sequence. As genomic alignments revealed that intron 1 of proglucagon is highly conserved across species, we cloned Venus in place of the coding region of proglucagon, between the start codon in exon 2 and the stop codon in exon 6. This region was initially replaced by a counter-selection cassette rpsL-neo (Genebridges) and subsequently this cassette was replaced by the YFP-Venus sequence. Briefly, the rpsL-neo or YFP-Venus sequences were amplified by PCR adding proglucagon gene specific 3' and 5' sequences (see oligonucleotides tabulated below) and homologous recombination was achieved upon co-transforming the BAC containing E.coli DH10B clone with the PCR product and the plasmid pSC101-BAD-gbaA, which provides the recombination enzymes (Genebridges). Positive recombinants were isolated using appropriate antibiotic selection and characterised by PCR and restriction analysis. Identity and correct positioning of the introduced YFP-Venus sequence was confirmed by direct sequencing. Three distinct BAC constructs were used to make the transgenic mice, one derived from a rat BAC, and two from a mouse BAC. Direct sequencing of the final constructs revealed that each mouse BAC had a nucleotide change resulting from the cloning strategy: one in the non-coding sequence in exon 2 (mBAC-v23), and the other in intron 1 (mBAC-v50). As neither mutation introduced a premature start codon or was predicted to interfere with splicing, both constructs were used to make transgenic mice. BAC-DNA for microinjection was purified using the large-construct Maxi-Prep kit (Qiagen) and dissolved at ~ 1-2 ng/µl in injection buffer containing (mM): 10 Tris-HCl pH 7.5, 0.1 EDTA, 100 NaCl, 0.03 spermine, 0.07 spermidine. Pronuclear injection into ova derived from C57B6/CBA F1 parents and reimplantation of embryos into pseudopregnant females was performed by the Central Biomedical Services at Cambridge

University. DNA of pups was isolated from ear clips by proteinase K digestion and screened for the transgene by PCR using the following primer pairs: rpg005/006, GLU003/004, GLU008/GFP001, GFP002/003 (and RM41/42, which amplifies β-catenin sequence used as a DNA quality control). Transgene copy number was estimated by RT-PCR comparing CT numbers for a transgene specific probe (YFP-forw, -rev and -probe) and Kir6.2 (Kcnj11-forw, -rev and -probe). The same probes were used with a ΔΔCT method to identify homozygous offspring after back-crossing into C57B6J for at least 7 generations. Five transgenic strains were established (estimated transgene copy number): rGLUE (2), mGLU-V23-099 (7), mGLU-V23-124 (12), mGLU-V50-144 (8) and mGLU-V50-145 (1).

Islet Isolation and FACS Sorting

Mice were killed by cervical dislocation and the pancreas injected immediately with 5 ml of collagenase V (1 mg/ml) in Ca^{2+} and Mg^{2+}-free HBSS. Pancreata were then dissected from the surrounding tissue and transported on ice. Following a 20 min digestion at 37°C, the pancreas was disrupted by vigorous shaking, and the islets picked and re-picked manually into RPMI containing 10% FBS. Islets were disrupted into single cells by trituration following a 2 min incubation in Ca^{2+}-free HBSS containing 0.1x trypsin/EDTA and 0.1% fatty acid-free bovine serum albumin (BSA). Cells were centrifuged at 300 g, re-suspended in supplemented RPMI and immediately sorted by flow cytometry. For islet sorting, we further subdivided non-fluorescent cells into a population that were Venus negative, larger (according to side and forward scatter) and with high background autofluorescence at 530 and 580nm, and a third population that were Venus negative, smaller and with low background autofluorescence.

Mouse pancreatic islets were cultured in RPMI on glass coverslips for 48 hours. Islets and cultured colonic cells were fixed in 4% paraformaldehyde (PFA) for 30 mins and stored in 0.1% PBS-sodium azide at 4˚C until needed. Immunostaining was performed as in methods

Immunohistochemistry Methods

Cultured colonic cells were fixed in 4% paraformaldehyde (PFA) for 30 mins and stored in 0.1% PBS-sodium azide at 4˚C until needed. Freshly isolated mouse ileum and colon were fixed with PFA for 48 hours. Tissues were cryoprotected in 20% sucrose for 48 hrs and embedded in OCT prior to sectioning. Tissue sections (8 µm) and whole islets were blocked in 10 % goat serum for 1 hr and incubated with glucagon antibody (1:300, Santa Cruz Biotechnology, Inc.) for 3 hrs at room temperature, or with PYY antibody (1:100, Progen, Germany) overnight at 4˚C. Tissues were then incubated for 1 hr at room temperature with Alexa 635- (1:500), Alexa 555- (1:1000) conjugated goat anti-rabbit secondary antibodies (Invitrogen, UK), or with Alexa 633-conjugated goat anti-guinea pig secondary (1:300, Invitrogen, UK), as appropriate. Tissue samples stained with secondary antibody alone served as controls. Images were captured using a Zeiss LSM 510 META confocal microscope (Carl Zeiss, UK).

For SGLT1 staining of ileal sections an antigen retrieval step was required to recover the antigenic sites masked by paraformaldehyde fixation. This entailed heating the sections at 125 °C for 3 min in tri-sodium citrate buffer (10 mM, pH 6). The poly-L-lysine coated slides, onto which the tissue sections were mounted, were allowed to cool and subsequently rinsed with phosphate-buffered saline. Sections were blocked as described above and incubated at room temperature with an SGLT1 antibody (Mace et al., 2007) used at a dilution of 1:100. Owing to the loss of Venus fluorescence as a consequence of the antigen retrieval step, a fluorescein isothiocyanate-conjugated GFP antibody (1:100, Abcam) was also added for a 3 hour period (room temperature) to label the L-cells. To evaluate the specificity of the SGLT1 antibody, an excess of antigenic peptide was incubated with the antibody prior to tissue staining, as described previously (Mace et al., 2007).

EdU Staining

Cell proliferation was assessed using the Click-iT™ EdU assay kit (Invitrogen, UK). Cells isolated from mouse colon were cultured for a 3 day period prior to replacing growth media with fresh complete media supplemented with the nucleoside analog 5-ethynyl-2'-deoxyuridine (EdU, 10μM). Cells were incubated in the presence of EdU for 24 hours and subsequently fixed with 3.7% PFA. The remaining steps of this assay were performed in accordance with the manufacturer's guidelines.

Immunofluorescence Microscopy of Cultured Cells

Cells grown on matrigel-coated glass-bottom culture dishes (Mattek Corporation, USA) for an 8-day period were fixed with 4% PFA for 30 minutes at room temperature and subsequently incubated for 1 hour in PBS (containing 0.1%, vol/vol, Triton X-100) supplemented with 10% (vol/vol) goat serum. Cells were then incubated for 3 hours at room temperature with either a PYY-, glucagon- or SGLT-1 primary antibody, each used at a dilution of 1: 100. After three 5 minute washes with PBS, cells were incubated with the appropriate Alexa 633-conjugated secondary antibody for 1 hour (room temperature). Finally, the cells were rinsed three times in PBS for 5 minutes and then covered with a glass coverslip. Control experiments were performed in parallel but with the primary antibody omitted. All of the presented images were captured using a Zeiss LSM 510 META confocal microscope (Carl Zeiss, UK).

Collection of Tongue Epithelium from Circumvallate Region

The epithelium of the tongue was separated by collagenase digestion, and the circumvallate region dissected using a stereomicroscope. mRNA was extracted as described in the methods for the treatment of FACS sorted cells.

Supplementary Figure Legends

Figure S1

A. The BAC construct for making transgenic mice was made by cloning Venus into the coding region of proglucagon (for further details see supplementary methods).
B. Colocalisation of direct Venus fluorescence (green) with PYY immunofluorescence (red) in a fixed colonic slice. The scale bar represents 10 μm.
C. Immunostaining for Venus (green) and SGLT1 (red) in a fixed ileal slice, showing the apical localisation of SGLT1 on the villus. The scale bar represents 10μm.
D. Immunostaining for glucagon or PYY in 8-day old colonic cultures. Red, glucagon or PYY (as indicated); green, direct Venus fluorescence; blue, DAPI. DIC, Venus and DAPI overlays were generated using a 63x objective lens whereas the remaining images were generated using a 100x objective. All scale bars represent 20 μm.
E. EdU incorporation (red) in 3-day old colonic cultures incubated for a further 24 hours in EdU. Blue, Hoechst; green, direct Venus fluorescence. Scale bar represents 20 μm.
F. Immunostaining for SGLT1 in 8-day old colonic cultures. Red, SGLT1; green, direct Venus fluorescence. Scale bar represents 20 μm.

Figure S2. Expression of Venus in Islet Cells from Transgenic Mice

A. Colocalisation of direct Venus fluorescence (green) with glucagon immunofluorescence (red) in pancreatic islets. The scale bar represents 20 μm.
B. Single cells were selected by flow cytometry according to their pulse width vs forward scatter characteristics (not shown), and by their yellow/green fluorescence (left) and forward/side scatter (right). Cell population 1 was collected by gating for Venus fluorescence in the yellow/green channels (green data points). Cell population 2 was collected by gating for a high background autofluorescence in the yellow/green channels (red data points) and higher forward and side scatter. Cell population 3 was collected by gating for a lower background autofluorescence (blue data points), and lower forward and side scatter. Cells in the 3 different gates on the yellow/green axes are labelled by the corresponding colours in the forward/side scatter plot.
C. Expression of glucagon, insulin, somatostatin and pancreatic polypeptide in the three cell populations (1, green; 2, red; 3, blue) collected as in A and analysed by quantitative RT-PCR. Expression was normalised to that of β-actin in the same sample. Data represent samples from three sorts (each with islets pooled from 3-4 mice) for populations 1 and 2, and two sorts for population 3. Data are presented as geometric mean, and the error bar was calculated from the log(base 2) data.

Figure S3. Sucralose Pretreatment

Mixed colonic cultures were supplemented overnight with 20 mM sucralose (grey bars) or cultured in control unsupplemented media (white bars) and stimulated the next day in standard bath solution containing 10 mM glucose (gluc), or 10 mM glucose plus 20 mM sucralose, as indicated. GLP-1 release is expressed relative to the basal secretion in either untreated or sucralose-pretreated control wells, as appropriate. Basal secretion levels were 3.6% and 3.0% in untreated and sucralose-pretreated cells, respectively (not significantly different). Error bars represent 1 SE, n=3 samples each from a single experiment. Significance is shown relative to baseline using a one-sample t-test * $p<0.05$, ** $p<0.01$; or by comparing columns as indicated, Δ $p<0.05$, ΔΔ $p<0.01$.

Suppl fig 1

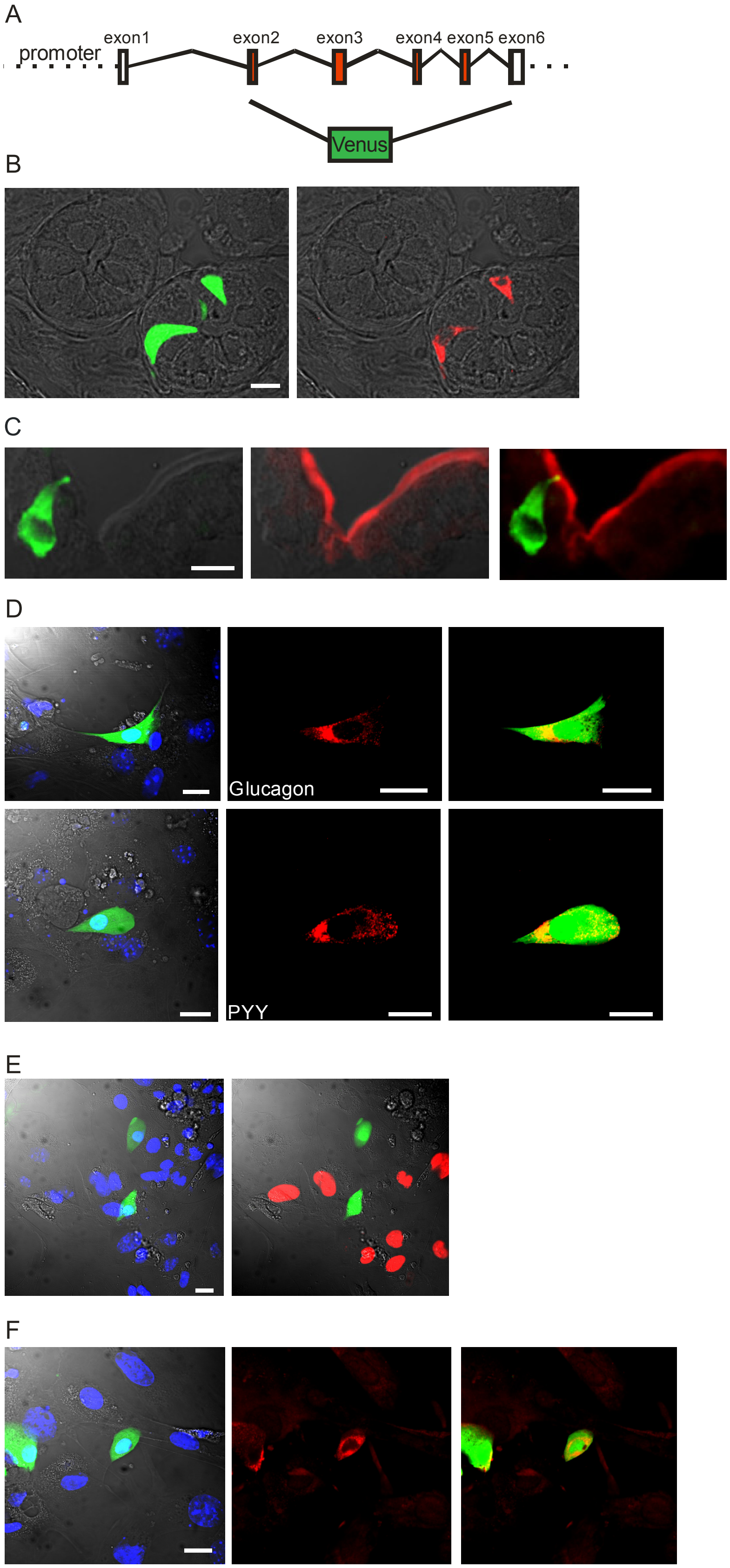

A
promoter
exon1
exon2
exon3
exon4 exon5 exon6
Venus
B
C
D
Glucagon
PYY
E
F

suppl figure 2

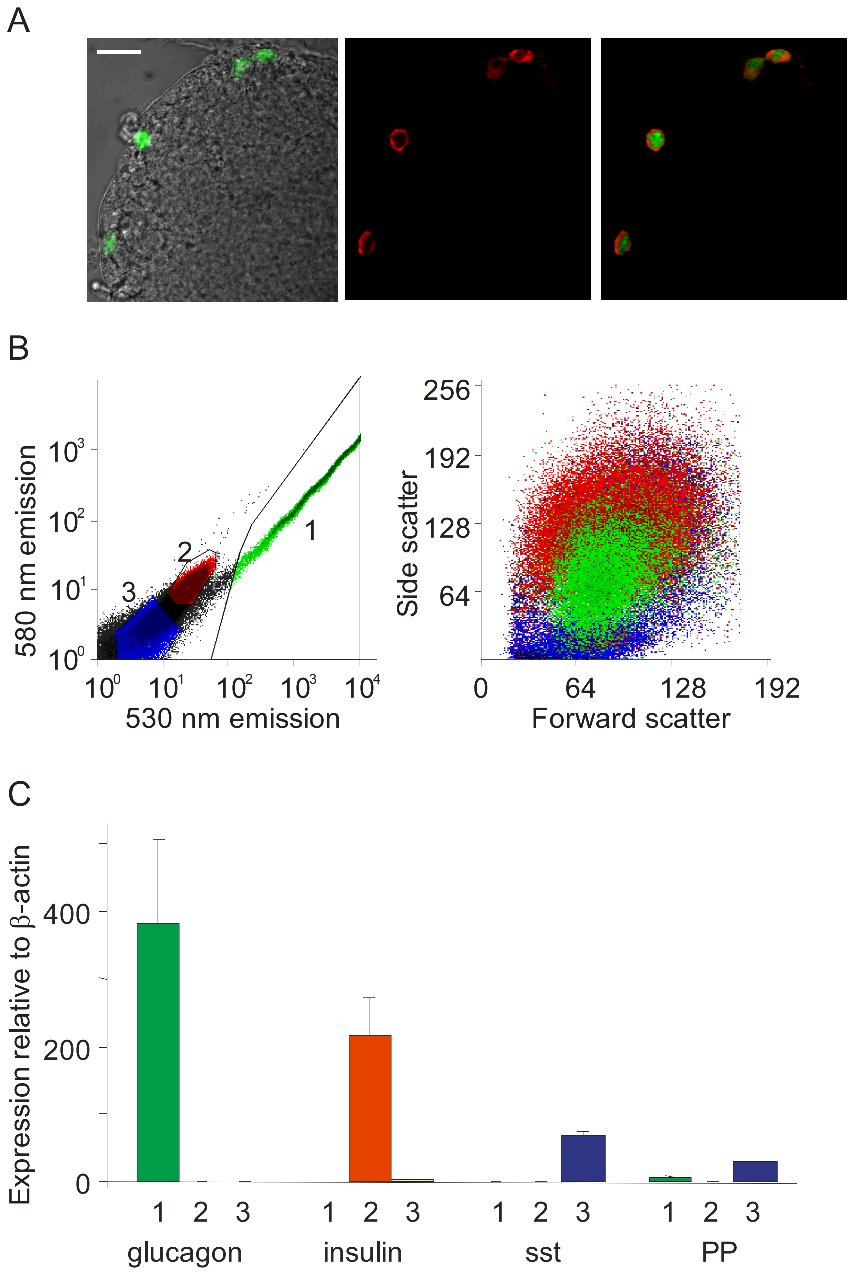

Suppl fig 3

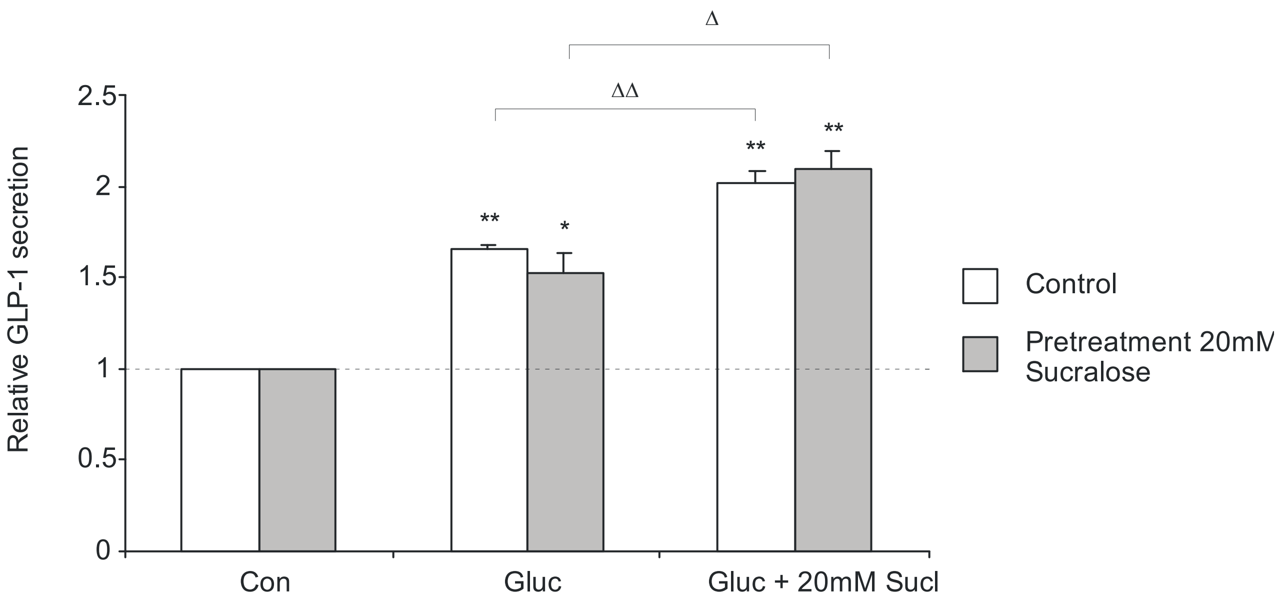
Δ
ΔΔ
Relative GLP-1 secretion
2.5
2
1.5
1
0.5
0
** * ** **
Con
Gluc
Gluc + 20mM Sucl
Control
Pretreatment 20mM
Sucralose

Table S1: ***Expression Data from L_{pos}, L_{neg}, Islet Cells and Enteroendocrine Cell Lines***

Expression was analysed by quantitative RT-PCR and expressed as a ΔCT compared with β-actin in the same sample. Data are presented as mean ± SE, with n≥3 samples from separate mice for each value, except in the case of δ/PP cells, where n=2 (and therefore no SE). (<) marks data where fewer than 2 samples gave a detectable reading, indicating that the expression level is less than the value indicated. Expression in L_{pos} and L_{neg} cells from the same intestinal region were compared by Student's t-test; * p<0.05, ** p<0.01, *** p<0.001.

			Small intestine, mid third		Small intestine, lower third		Colon		Pancreatic islets		
**Gene**	**GLUTag**	**STC-1**	**L_{pos}**	**L_{neg}**	**L_{pos}**	**L_{neg}**	**L_{pos}**	**L_{neg}**	**α**	**β**	**δ/PP**
Kir6.2	-6.5±0.2	-7.7±0.1	-3.6±0.4*	-10.3±2.0 (<)	-3.6±1.0**	-11.4±1.4 (<)	-3.0±0.8***	-10.4±1.2	-2.4±0.2	-1.6±0.3	-2.0
SUR1	-4.1±0.1	-4.3±0.4	-2.0±0.7**	-10.1±1.2	-1.8±0.9**	-11.5±1.5 (<)	-0.4±0.5***	-8.7±0.5	0.4±0.3	1.1±0.3	0.7
GK	-5.2±0.2	-4.4±0.3	-4.2±0.9**	-12.3±1.0 (<)	-4.4±1.0**	-12.1±1.2 (<)	-4.6±0.3**	-11.7±1.6 (<)	-4.6±0.4	-3.5±0.3	-4.4
SGLT1	-5.6±0.2	-10.8±0.5	-2.2±0.5	-1.6±0.3	-2.2±0.1	-3.2±0.8	-1.7±0.4**	-5.5±0.5	-15.5±0.2 (<)	-10.9±0.9 (<)	-11.1
GLUT1	-5.0±0.5		-7.9±0.5	-7.3±0.2	-6.6±0.4	-7.3±0.4	-4.6±0.4	-4.9±0.1	–6.9±0.7	-6.1±0.2	-5.7
GLUT2	-14.7±1.3		-6.5±1.2	-5.0±0.7	-9.3±1.0	-8.4±0.5	-11.2±0.7	-12.7±1.4 (<)	-10.4±0.5	-0.2±0.3	-6.3
GLUT3	-3.9±0.5		-11.6±1.1	-10.3±0.5	-12.3±1.0 (<)	-10.8±1.2	-13.9±1.4 (<)	-10.1±0.4	-6.7±0.5	-10.7±1.1	-7.0
GLUT5	-2.8±0.3		-4.0±0.3	-4.3±0.2	-4.1±0.3	-5.8±0.7	-3.2±0.2***	-9.3±0.4	-2.4±0.2	-0.2±0.2	-1.9
		**Tongue**									
Tas1R1	-16.0±0.5	-14.3±0.3	-15.0±0.8 (<)	-12.9±0.5 (<)	-13.7±1.8 (<)	-12.5±1.5 (<)	-13.5±0.7 (<)	-16.1±0.6 (<)	-13.4±1.0	-15.5±0.6 (<)	-14.5 (<)
Tas1R2	-16.2±0.1	-17.2±0.5	-15.0±0.8 (<)	-12.9±0.5 (<)	-11.3±1.0	-12.5±1.5 (<)	-11.6±0.9*	-15.4±0.8 (<)	-12.9±1.3	-16.0±1.1 (<)	-13.8 (<)
Tas1R3	-11.3±0.7	-11.1±0.4	-8.7±0.8	-9.6±1.3	-9.1±0.4	-10.6±1.1	-8.3±0.5	-9.7±0.3	-7.6±0.1	-7.5±0.7	-8.2
Gustducin	-16.4±0.6	-11.7±0.4	-16.2±0.8 (<)	-13.7±1.0 (<)	-14.1±1.1 (<)	-12.7±1.9 (<)	-12.0±0.6	-10.3±0.8 (<)	-10.8±0.7	-15.9±2.2 (<)	-10.1

Table S2: ***PCR Primers***

Name	Sequence
FRGLU001	TTC CCC ATC ATC CCC CTA CCC CCC ACT CTG TGT TCC AAC AGG CAG AAT AAA AAA ATG GCC TGG TGA TGA TGG CGG GAT CG
FRGLU002	CAA GTG ACT GGC AGG AGA TGT TGT GAA GAT GGT TGT GAA TGG TGA AAT ATT CCT ATC AGA AGA ACT CGT CAA GAA GGC G
FRGLU004	TGG TGC AGA AGG GCA GAG C
FRGLU005	CCT TAC AGT CCT GGT AAT GGA C
FRGLU005	CAT CCC AAG TGA CTG GCA GGA GAT GTT GTG AAG ATG GTT GTG AAT GGT GAA ATA TTC CTA CTT GTA CAG CTC GTC CAT GCC GAG
FRGLU006	TGC TTC CCC ATC ATC CCC CTA CCC CCC ACT CTG TGT TCC AAC AGG CAG AAT AAA AAA TCC ACC ATG GTG AGC AAG GGC GAG
FRGLU007	CCG CAT GCA AAG CAG TAT AGC
FRGLU008	AAT TGA GCT CAT TTG GAC TGC C
GFP001	CTT GCC GTA GGT GGC ATC G
GFP002	CTG GTA GTG GTC GGC GAG C
GFP003	GTT CAG CGT GTC CGG CGA G
mGLP001	TAC ATC CCA AGT GAC TGG CAC GAG ATG TTG TGA AGA TGG TTG TGA ATG GTG AAA TAC CTA TCA GAA GAA CTC GTC AAG AAG GCG
mGLP002	TAC ATC CCA AGT GAC TGG CAC GAG ATG TTG TGA AGA TGG TTG TGA ATG GTG AAA TAC CTA CTT GTA CAG CTC GTC CAT GCC GAG
mGLP003	TCC CCC ATC ACC CCC ATC CCA CCC CCA TTC TGT GTT CCA TCA GGC AGA AAA AAA AAT GGC CTG GTG ATG ATG GCG GGA TCG
mGLP005	CAT CTG CAT GCA AAG CAA TAT AGC
mGLP006	TGC TCC CCC ATC ACC CCC TAC CCA CCC CCA TTC TGT GTT CCA TCA GGC AGA AAA AAA ATC CAC CAT GGT GAG CAA GGG CGA G
mGLP007	GCA AGG CTA AAC AGC CTG GAG
rpg005	CAC TCT ACA CAT TGA AGC ACA ATC G
rpg006	GCT CAG GTC TGG TTT ATG GAA TCA G
RM41	AAG GTA GAG TGA TGA AAG TTG TT
RM41	CAC CAT GTC CTC TGT CTA TTC
YFP-forw	GCA AAG ACC CCA ACG AGA A
YFP-rev	GGC GGC GGT CAC GAA
YFP-probe	6-FAM-CGC GAT CAC ATG GTC CTG CTG-TAMRA
mKcnj11-fw	CCC GCT TCG TGT CCA AGA
mKcnj11-rev	CAG CGT GGT GAA CAC ATC CT
mKcnj11-probe	6-FAM-CAA CGT CGC CCA CAA GAA CAT TCG A-BHQ-1

Table S3: ***Quantitative RT-PCR Primers and Probes***

Gene	Gene alias	Forward	Reverse	Probe
kcnj11	Kir6.2	ccc gct tcg tgt cca aga	cag cgt ggt gaa cac atc ct	caa cgt cgc cca caa gaa cat tcg a
Abcc8	SUR1	acc atc ttg tac ctt tgc tta ttg aa	tag cct acg cat ctc aga aac ca	ttc tcc tcc acc gtg gca ttt agc a
Gck	GLK	atg tga ggt cgg cat gat tgt	cct tcc acc agc tcc aca tt	cac cgg ctg caa cgc ctgc
Slc5a1	Sglt1	tgg tgt acg gat cag gtc attg	ttc aga tag cca cac agg gta cag	cga tgc ctc tcg gcc aag aac atg
Slc2a1	Glut1	ggt atc aat gct gtg ttc tac tac tca a	cca cag tga agg ccg tgt t	tac gcc acc atc ggc tcc ggt at
Slc2a2	Glut2	tgg ctg cct tca gca actg	caa gga agt ccg caa tgt act g	ctg caa ttt tgt cat cgc cct ctg ctt
Slc2a3	Glut3	gag gag aac cct gca tat gat agg	caa agc tca tgg ctt cat agt ca	cat ggc tgt ttg ctc cgt ttt cat gac
Slc2a5	Glut5	tca tga cca tcc tca cga tct tt	gcg gcc gtc agc act aag	acg gcg att cct act cct cgt cgg
Tas1r1	T1r1	tga cac agg cac ctg att gtt c	tct gcc tca ctg tct cca gtt c	tcc cgt gcc act gga ttt ttg tct tat tt
Tas1r2	T1r2	gtt cgt cga aga aga gct ggt t	gtc cgc tgc acc aag ca	cca gat ctc cct gag tag ctg cca tgg
Tas1r3	T1r3	tca gag ctt gcc ctc att aca g	tgt gcg gaa gaa gga tgg a	ttc ctc atg cca cag gtc agc tat agt gc
Ffar1	GPR40	cgc tgg gct ttc cat tga	gct ggg agt gag tcg cag tt	cca tcc gag gcg cag tgt ccc
Gpr120	Gpr120	gtc aca aaa aac aca tcc cat ga	ggg acc agg aaa ttc cga tt	tct cct atg cgg ttg ggc caa tcc
Gpr119	Gpr119	cca ctc gga gtc tcc ata ttc c	acc ttg ggt gaa aca cag caa	cag acc acc tac cat gga ccc tgc a

The TaqMan probes for Actb (β-actin), Gcg (proglucoagon), Gpbar1 (TGR5) and Pyy and α-gustducin were purchased from Applied Biosystems. Quantitative primer pairs were tested against a concentration series of cDNA to confirm that they diluted linearly.
